# Supplementary material for: Invasive European green crab (Carcinus maenas) predation in a Washington State estuary revealed with DNA metabarcoding
Source: PLoS One. 2024 May 31;19(5):e0302518. doi: 10.1371/journal.pone.0302518 (PMC11142710; doi:10.1371/journal.pone.0302518)
Supplement: S1 Text — (DOCX) [file pone.0302518.s001.docx]

### Trapping Protocol

Galvanized steel minnow traps (Gee’s G-40), and square Fukui fish traps were baited with Pacific mackerel (*Scomber japonicus*) and set for the nighttime high tide. At clam bed sites, traps were arrayed in 5 horizontal transects of 6 traps each, alternating trap type. Traps within each horizontal transect, and each of the five horizontal transects, were separated by approximately 20m, such that the array formed a checkerboard grid. In natural sloughs, 10 traps of each type were set in the middle of the slough following the main channel, starting at the highest elevation at which halophyte vegetation was apparent on the shore, and extending down channel, alternating trap type, again with each trap separated by 20m. On trap retrieval, all organisms in each trap were recorded to species, and a subset of all crab species were measured (carapace width) to the nearest mm. Native species were released, with the exception of Dungeness crabs (*Cancer magister*) which were retained for another project.
