# Supplementary material for: Invasive European green crab (Carcinus maenas) predation in a Washington State estuary revealed with DNA metabarcoding
Source: PLoS One. 2024 May 31;19(5):e0302518. doi: 10.1371/journal.pone.0302518 (PMC11142710; doi:10.1371/journal.pone.0302518)
Supplement: S2 Table — (DOCX) [file pone.0302518.s003.docx]

Table S2. Mock community composition for samples in which species were present in even (A-C) and skewed proportions (D-E), with the DNA (ng) per species added to each community in the final column. Skewed proportions, except for green crab in community D, were created by multiplying 20ng by an ‘adjustment factor’ between 0.2 and 2.0, generated using the “RANDBETWEEN” function in Microsoft Excel. The adjustment factor for green crab in Sample D was set manually to ensure that the relative amount of green crab DNA in the sample would be approximately 2-3x the amount of DNA from any other species (intended to replicate over-representation of predator DNA in a stomach content sample).

| **Label** | **Descriptor** | **Species** | **Relative proportion** | **DNA per species (ng)** |
| --- | --- | --- | --- | --- |
| A | All species, even proportions | *C. maenas, R. philippinarum, M. arenaria, C. magister, H. oregonensis, C. franciscorum, B. attramentaria, Z. marina, C. aggregata, L. armatus, C. asper* | 1 | 20 |
| B | No green crab, even proportions | *R. philippinarum, M. arenaria, C. magister, H. oregonensis, C. franciscorum, B. attramentaria, Z. marina, C. aggregata, L. armatus, C. asper* | 1 | 20 |
| C | No crab or shrimp, even proportions | *R. philippinarum, M. arenaria, B. attramentaria, Z. marina, C. aggregata, L. armatus, C. asper* | 1 | 20 |
| D | All species, skewed proportions | *C. maenas* | 3.3 | 66 |
|  |  | *R. philippinarum* | 0.88 | 17.60 |
|  |  | *M. arenaria* | 1.01 | 20.20 |
|  |  | *C. magister* | 0.30 | 6 |
|  |  | *H. oregonensis* | 0.45 | 9 |
|  |  | *C. franciscorum* | 0.77 | 15.40 |
|  |  | *B. attramentaria* | 0.74 | 14.80 |
|  |  | *Z. marina* | 0.98 | 19.60 |
|  |  | *C. aggregata* | 1.10 | 22 |
|  |  | *L. armatus* | 0.93 | 18.60 |
|  |  | *C. asper* | 0.95 | 19 |
| E | No green crab, skewed proportions | *R. philippinarum* | 1.60 | 32 |
|  |  | *M. arenaria* | 1.20 | 24 |
|  |  | *C. magister* | 0.30 | 6 |
|  |  | *H. oregonensis* | 0.45 | 9 |
|  |  | *C. franciscorum* | 1.50 | 30 |
|  |  | *B. attramentaria* | 1.09 | 21.80 |
|  |  | *Z. marina* | 1.80 | 36 |
|  |  | *C. aggregata* | 1.70 | 34 |
|  |  | *L. armatus* | 1.40 | 28 |
|  |  | *C. asper* | 0.65 | 13 |
