# Supplementary material for: Invasive European green crab (Carcinus maenas) predation in a Washington State estuary revealed with DNA metabarcoding
Source: PLoS One. 2024 May 31;19(5):e0302518. doi: 10.1371/journal.pone.0302518 (PMC11142710; doi:10.1371/journal.pone.0302518)
Supplement: S3 Table — (DOCX) [file pone.0302518.s004.docx]

Table S3. Mean and median estimated proportions of DNA contributed by calibrated prey species to an “average” crab diet, with 95% credible intervals. Proportions are provided across all sites, and then for each individual site type (clam bed sites = Oysterville, Long Beach; slough sites = Nahcotta, Stackpole).

| **Group** | **Species** | **Common name** | **Mean** | **Median** | **95% CI** |
| --- | --- | --- | --- | --- | --- |
| Clam bed | *Crangon franciscorum* | sand shrimp | 0.140 | 0.139 | (0.111,0.171) |
|  | *Leptocottus armatus* | Pacific staghorn sculpin | 0.129 | 0.129 | (0.104,0.159) |
|  | *Hemigrapsus oregonensis* | hairy shore crab | 0.126 | 0.125 | (0.101,0.155) |
|  | *Ruditapes philippinarum* | Manila clam | 0.123 | 0.123 | (0.113,0.133) |
|  | *Cymatogaster aggregata* | shiner perch | 0.123 | 0.122 | (0.098,0.151) |
|  | *Cancer magister* | Dungeness crab | 0.121 | 0.120 | (0.096,0.149) |
|  | *Batillaria attramentaria* | mud snail | 0.119 | 0.119 | (0.095,0.147) |
|  | *Mya arenaria* | soft-shell clam | 0.119 | 0.119 | (0.096,0.146) |
| Slough | *Hemigrapsus oregonensis* | hairy shore crab | 0.425 | 0.424 | (0.312,0.543) |
|  | *Crangon franciscorum* | sand shrimp | 0.324 | 0.322 | (0.241,0.412) |
|  | *Mya arenaria* | soft-shell clam | 0.252 | 0.249 | (0.162,0.355) |
| All | *Cymatogaster aggregata* | shiner perch | 0.148 | 0.147 | (0.119,0.181) |
|  | *Leptocottus armatus* | Pacific staghorn sculpin | 0.140 | 0.140 | (0.113,0.17) |
|  | *Cancer magister* | Dungeness crab | 0.123 | 0.122 | (0.098,0.15) |
|  | *Ruditapes philippinarum* | Manila clam | 0.122 | 0.122 | (0.112,0.132) |
|  | *Mya arenaria* | soft-shell clam | 0.122 | 0.121 | (0.098,0.149) |
|  | *Hemigrapsus oregonensis* | hairy shore crab | 0.117 | 0.116 | (0.094,0.143) |
|  | *Crangon franciscorum* | sand shrimp | 0.115 | 0.115 | (0.092,0.141) |
|  | *Batillaria attramentaria* | mud snail | 0.114 | 0.113 | (0.091,0.14) |
