# Supplementary material for: Invasive European green crab (Carcinus maenas) predation in a Washington State estuary revealed with DNA metabarcoding
Source: PLoS One. 2024 May 31;19(5):e0302518. doi: 10.1371/journal.pone.0302518 (PMC11142710; doi:10.1371/journal.pone.0302518)
Supplement: S5 Table — (DOCX) [file pone.0302518.s006.docx]

Table S5. Prey items found in green crab stomach contents that are also bycatch species. We provide the number of crab with the prey item in its stomach, the number of those crab which also co-occurred with the given prey item in the trap, and the percent of crab that did *not* co-occur with the given prey item in the trap. The crab at Long Beach which had *Hemigrapsus sp.* identified from its stomach contents co-occurred with one *Hemigrapsus oregonensis* in the trap.

| Prey taxa | Site | N Crab | N Crab **with** co-occurrence | Percent Crab **without** co-occurrence |
| --- | --- | --- | --- | --- |
| *Batillaria attramentaria* | Oysterville | 4 | 0 | 100 |
| *Hemigrapsus sp.* | Long Beach | 1 | 1 | 0 |
| *Hemigrapsus oregonensis* | Long Beach | 2 | 0 | 100 |
| *Hemigrapsus oregonensis* | Nahcotta | 1 | 0 | 100 |
| *Hemigrapsus oregonensis* | Oysterville | 3 | 3 | 0 |
| *Hemigrapsus oregonensis* | Stackpole | 6 | 1 | 83 |
| *Leptocottus armatus* | Long Beach | 4 | 1 | 75 |
| *Leptocottus armatus* | Oysterville | 2 | 0 | 100 |
| *Cancer magister* | Long Beach | 2 | 2 | 0 |
| *Cancer magister* | Oysterville | 1 | 1 | 0 |
| *Pholis gunnellus* | Oysterville | 1 | 0 | 100 |
