# Supplementary material for: Invasive European green crab (Carcinus maenas) predation in a Washington State estuary revealed with DNA metabarcoding
Source: PLoS One. 2024 May 31;19(5):e0302518. doi: 10.1371/journal.pone.0302518 (PMC11142710; doi:10.1371/journal.pone.0302518)
Supplement: S6 Table — (DOCX) [file pone.0302518.s007.docx]

Table S6. Permutational pairwise tests of diet composition between sites of the same type, with (“corrected p value”) and without (“p value”) Bonferroni correction (for six total pairwise tests per data set) applied to the p-value. Tests were conducted using (a) a presence/absence matrix, and (b) the eDNA index. Superscript indicates significance at the α=0.05 (*), and 0.01 (**) levels.

| **(a)** |  | **Degrees of Freedom** | **Sum of Squares** | **R^2^** | **F statistic** | **p value** | **Corrected p value** |
| --- | --- | --- | --- | --- | --- | --- | --- |
|  | *Oysterville v. Long Beach (clam bed sites)* | | | | | | |
|  | site | 1 | 0.542 | 0.030 | 1.171 | 0.258 | 1.548 |
|  | Residual | 38 | 17.585 | 0.970 |  |  |  |
|  | Total | 39 | 18.127 | 1.000 |  |  |  |
|  | *Nahcotta v. Stackpole (natural slough sites)* | | | | | | |
|  | site | 1 | 0.338 | 0.038 | 0.759 | 0.755 | 4.530 |
|  | Residual | 19 | 8.469 | 0.962 |  |  |  |
|  | Total | 20 | 8.807 | 1.000 |  |  |  |
| **(b)** |  |  |  |  |  |  |  |
|  | *Oysterville v. Long Beach (clam bed sites)* | | | | | |  |
|  | site | 1 | 0.598 | 0.033 | 1.278 | 0.154 | 0.924 |
|  | Residual | 38 | 17.773 | 0.967 |  |  |  |
|  | Total | 39 | 18.370 | 1.000 |  |  |  |
|  | *Nahcotta v. Stackpole (natural slough sites)* | | | | | |  |
|  | site | 1 | 0.284 | 0.031 | 0.609 | 0.923 | 5.538 |
|  | Residual | 19 | 8.861 | 0.969 |  |  |  |
|  | Total | 20 | 9.145 | 1.000 |  |  |  |
