# Supplementary material for: Invasive European green crab (Carcinus maenas) predation in a Washington State estuary revealed with DNA metabarcoding
Source: PLoS One. 2024 May 31;19(5):e0302518. doi: 10.1371/journal.pone.0302518 (PMC11142710; doi:10.1371/journal.pone.0302518)
Supplement: S7 Table — (DOCX) [file pone.0302518.s008.docx]

Table S7. Evaluation of sample dispersion around centroids with the PERMDISP, based on (a) a presence/absence matrix, and (b) the eDNA index.

| **(a)** |  | **DF** | **Sum of Squares** | **R2** | **F** | **Pr(>F)** |
| --- | --- | --- | --- | --- | --- | --- |
|  | Site | 3 | 0.017867 | 0.11975 | 2.5847 | 0.054 |
|  | Residual | 57 | 0.131340 | 0.88025 |  |  |
|  | Total | 60 | 0.149207 | 1 |  |  |
| **(b)** |  |  |  |  |  |  |
|  | Site | 3 | 0.004869 | 0.04082 | 0.8085 | 0.495 |
|  | Residual | 57 | 0.114412 | 0.95918 |  |  |
|  | Total | 60 | 0.119281 | 1 |  |  |
