# Supplementary material for: Invasive European green crab (Carcinus maenas) predation in a Washington State estuary revealed with DNA metabarcoding
Source: PLoS One. 2024 May 31;19(5):e0302518. doi: 10.1371/journal.pone.0302518 (PMC11142710; doi:10.1371/journal.pone.0302518)
Supplement: S8 Table — (DOCX) [file pone.0302518.s009.docx]

Table S8. The ranked relative abundance of each calibrated prey item by site type, according to median sequencing read abundance (“Read abundance”) and median proportion of DNA in the stomach content sample (“Calibrated DNA abundance”; see Figure S4). ꜛ Indicates an increase in rank, and ꜜ indicates a decrease in rank.

| Rank | Read abundance | Calibrated DNA abundance |
| --- | --- | --- |
| 1 | *C. franciscorum* | *C. franciscorum* |
| 2 | *L. armatus* | *L. armatus* |
| 3 | *C. aggregata* | *R. philippinarum* ꜛ |
| 4 | *M. arenaria* | *C. aggregata* ꜜ |
| 5 | *B. attramentaria* | *B. attramentaria* |
| 6 | *M. (Cancer) magister* | *M. (Cancer) magister* |
| 7 | *R. philippinarum* | *H. oregonensis* ꜛ |
| 8 | *H. oregonensis* | *M. arenaria* ꜜ |
