# Supplementary figures and images for: Invasive European green crab (Carcinus maenas) predation in a Washington State estuary revealed with DNA metabarcoding
Source: PLoS One. 2024 May 31;19(5):e0302518. doi: 10.1371/journal.pone.0302518 (PMC11142710; doi:10.1371/journal.pone.0302518)

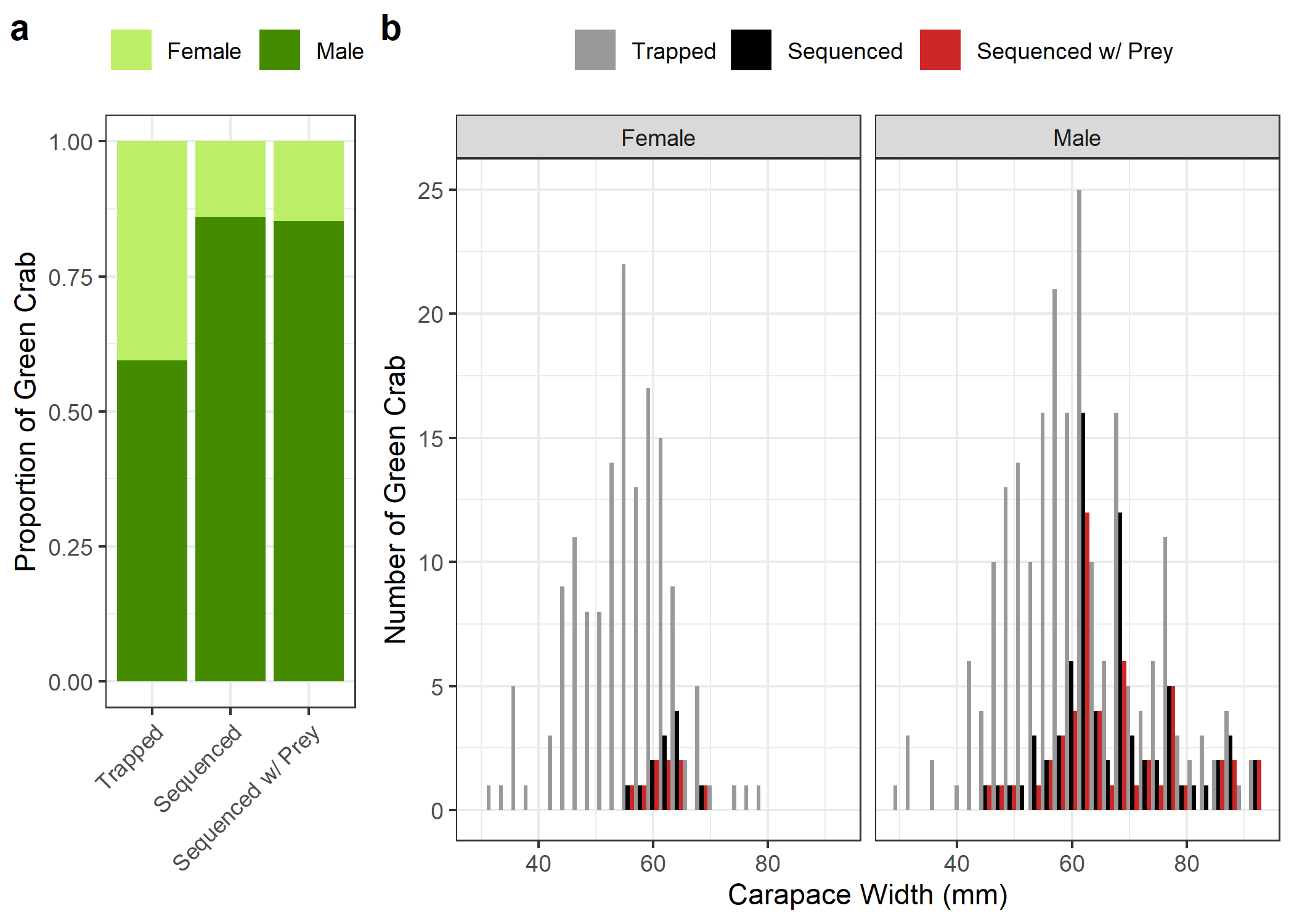

Supplement: S1 Fig — Sampling metadata according to sex, including (a) proportion of crab at each step in Fig 1B belonging to each sex, and (b) distribution of carapace widths. (PNG) [file pone.0302518.s010.png]

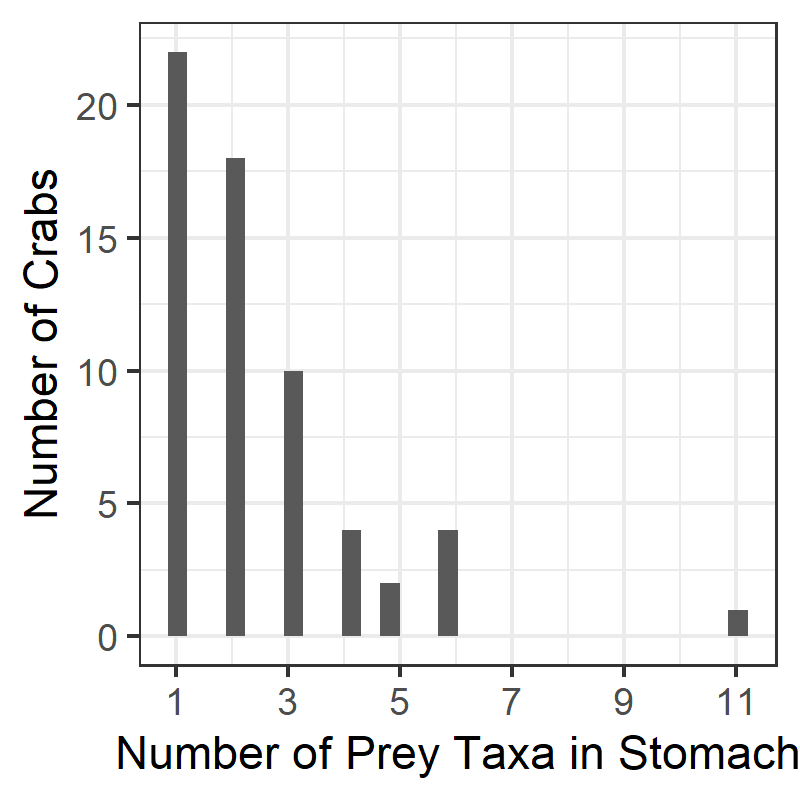

Supplement: S2 Fig — (PNG) [file pone.0302518.s011.png]

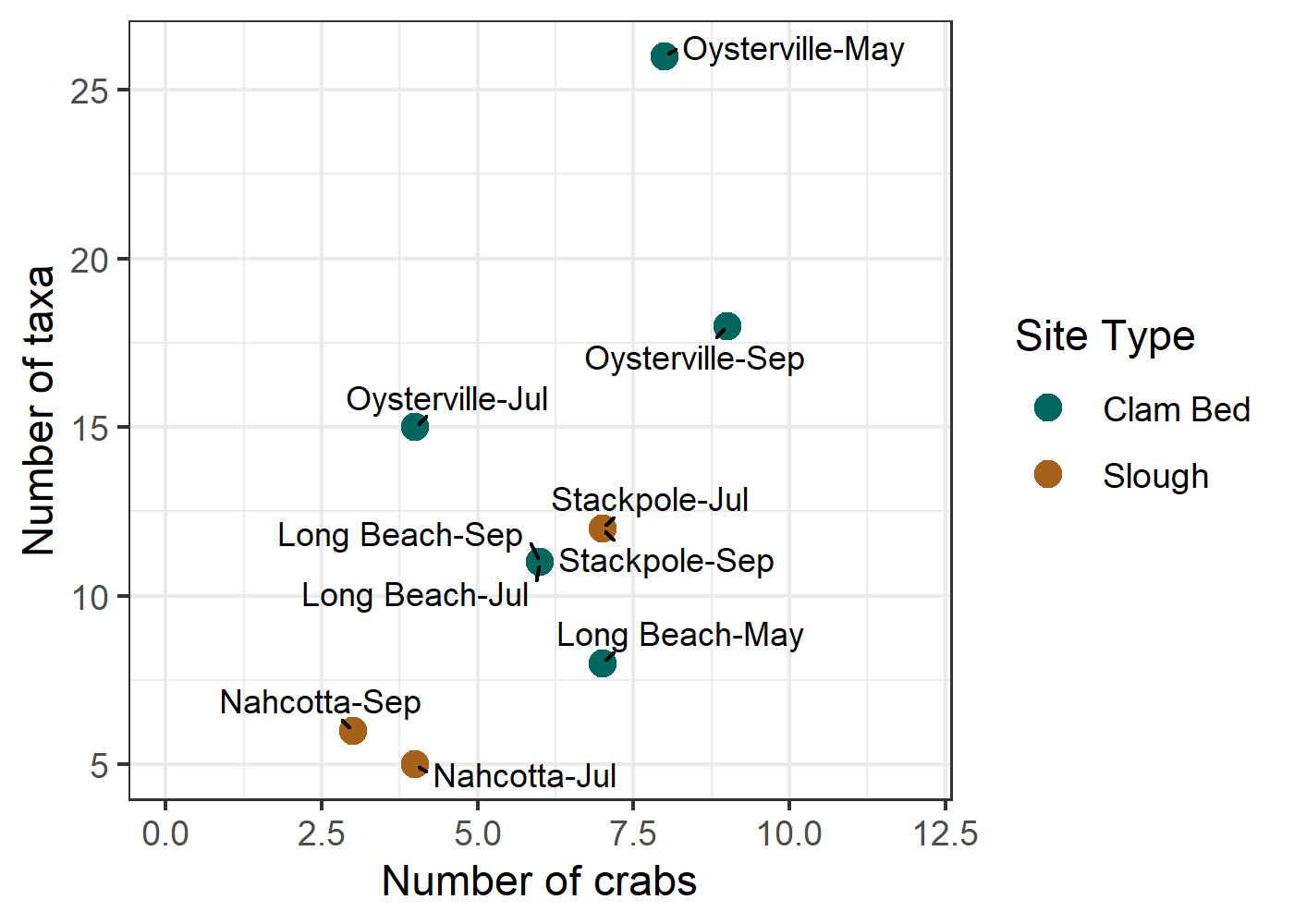

Supplement: S3 Fig — (PNG) [file pone.0302518.s012.png]

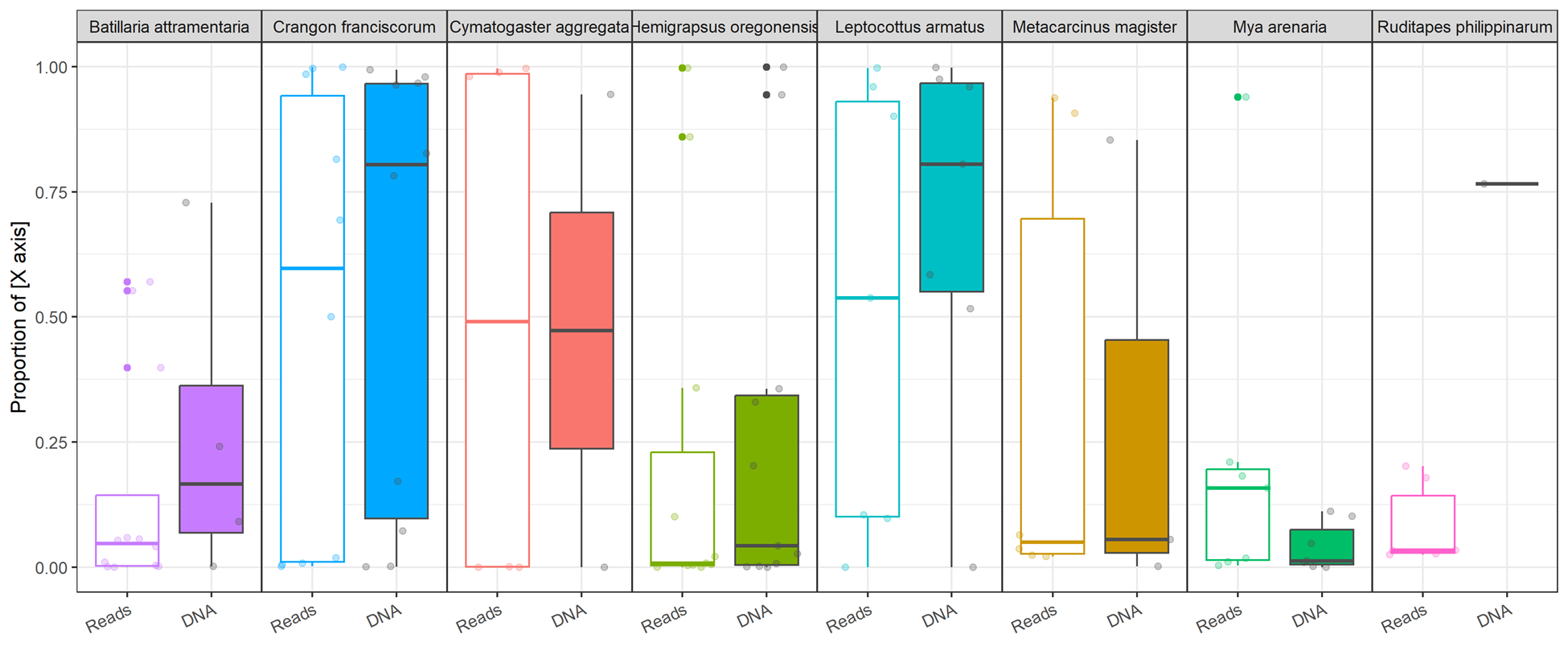

Supplement: S4 Fig — Differences in the distribution of the proportion of reads and proportion of DNA for each species are a result of accounting for amplification efficiencies using the Shelton et al. (2023) quantitative model. Partially transparent points represent individual observations; for reads, these are laboratory samples (the technical replicates of the crabs included in the analysis), and for DNA, these are crabs; for example, note that the Manila clam was detected in one crab, but we sequenced six technical replicates of that individual. When crabs with a given calibrated prey species had different numbers of technical replicates, we randomly sampled read proportions to the least number of technical replicates for crabs in that group. (PNG) [file pone.0302518.s013.png]

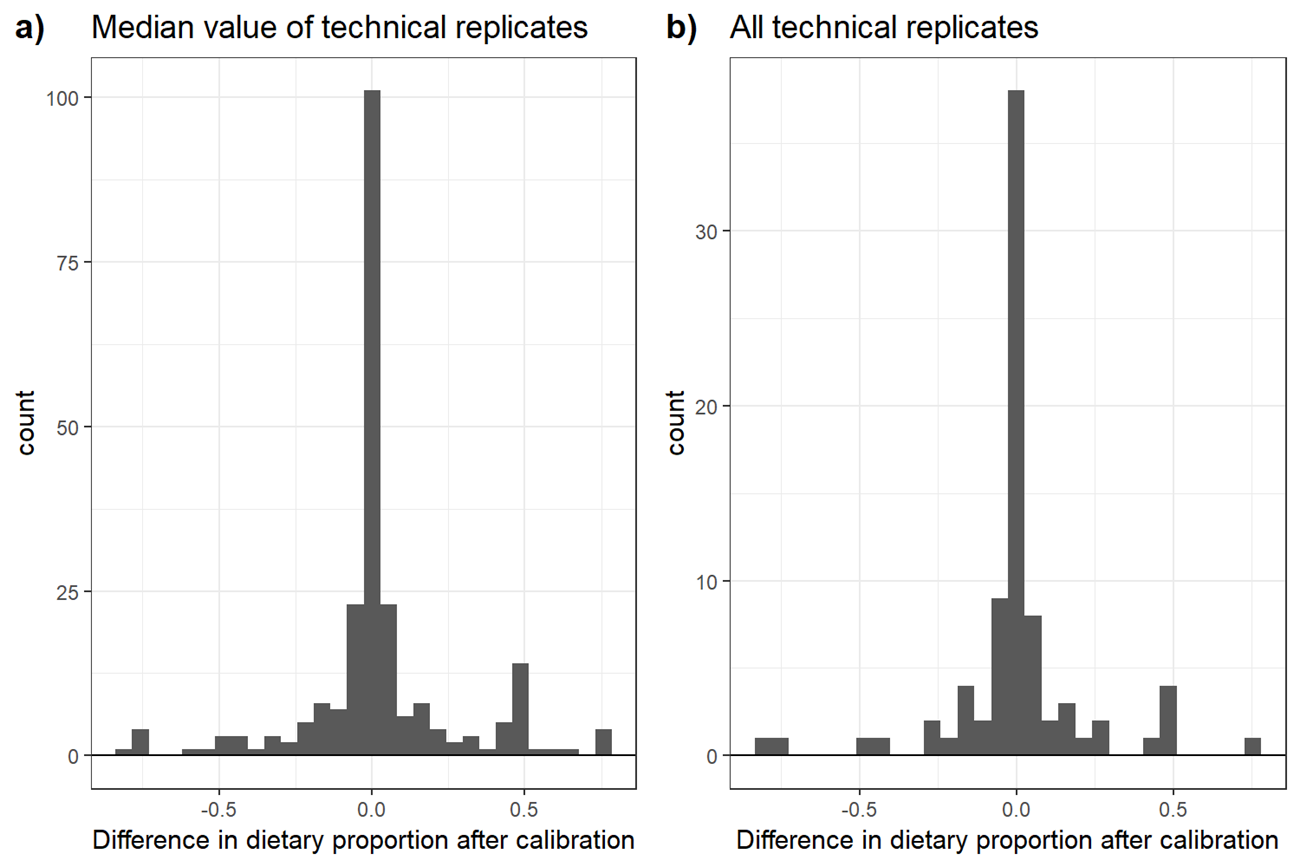

Supplement: S5 Fig — For each of the eight calibrated prey species, the observed proportion of sequencing reads (uncalibrated) was subtracted from the estimated proportion of DNA (calibrated), by either (a) using the median proportion of sequencing reads across all technical replicates for an individual crab, or (b) subtracting the estimated proportion of DNA from the observed proportion of sequencing reads for each individual technical replicate. (PNG) [file pone.0302518.s014.png]

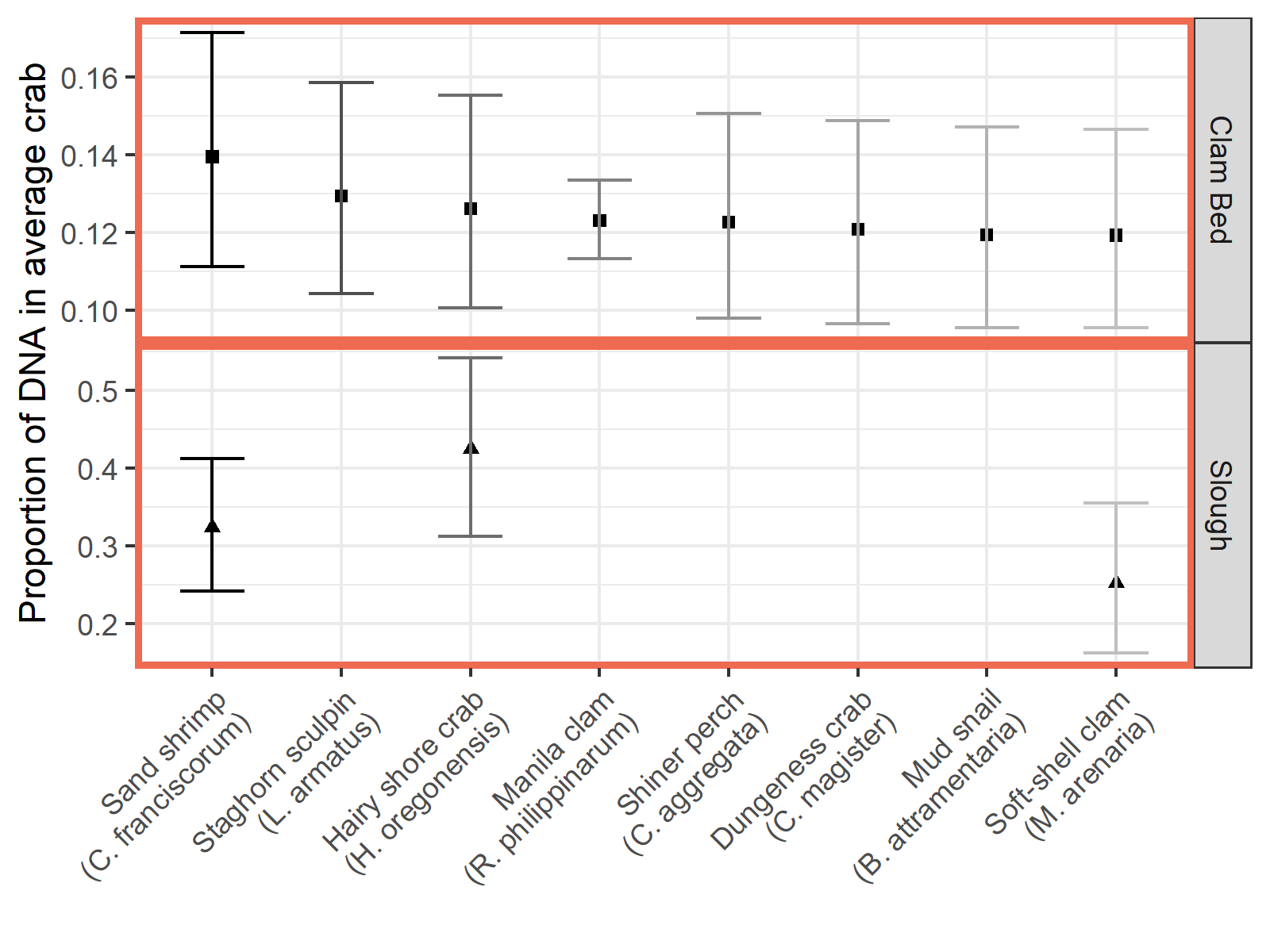

Supplement: S6 Fig — “Average” diet shown separately for a green crab from (a) clam bed sites and (b) slough sites. (PNG) [file pone.0302518.s015.png]
